# Nanogap Engineering for Enhanced Transmission of Wire Grid Polarizers in Mid-Wavelength Infrared Region

**DOI:** 10.1038/s41598-019-40614-6

**Published:** 2019-03-12

**Authors:** Wonyoung Kim, Minsuk Kim, Tae Young Kim, Hyunjin Choi, Myung-Jong Jin, Kyu-Tae Lee, Minbaek Lee, Chang Kwon Hwangbo

**Affiliations:** 1 Department of Physics, Inha University, 100 Inha-ro, Michuhol-gu, Incheon, 22212 Republic of Korea; 2 Agency for Defense Development, Daejeon, Republic of Korea; 3 Department of Chemistry and Chemical Engineering, Inha University, 100 Inha-ro, Michuhol-gu, Incheon, 22212 Republic of Korea

## Abstract

Wire-grid polarizers (WGPs) have been widely used in various fields, such as polarimetry, imaging, display, spectroscopy, and optical isolation. However, conventional WGPs used in diverse mid-wavelength infrared (MWIR) applications show high reflection losses, which intrinsically arise from high refractive indices of their IR-transmitting substrates, such as silicon (Si) and germanium (Ge). This study demonstrated the enhanced transmittance of a transverse magnetic (TM) wave that surpassed ~80% over the entire MWIR range from 3000 to 5000 nm in a narrow air gap of a WGP, where aluminum (Al) was selectively deposited on a nanopatterned Si substrate using an oblique angle deposition method. Moreover, a higher TM wave transmittance was achieved by reducing the air gaps of the WGPs in the nanopatterns, which were distinctly different from the traditional WGPs comprising metal wires patterned directly on a flat substrate. A finite-difference time-domain simulation was performed to investigate optical properties of the proposed WGPs, which showed that the electric field in the air nanogap was remarkably enhanced. The characteristic performances were further investigated using a combination of an effective medium approximation and an admittance diagram, revealing that the broadband transmission enhancement could be attributed to a combined effect of a strong electric field and a better admittance matching. The approach and results described in this paper hold promise for the design and the fabrication of high-quality WGPs, as well as their numerous applications.

## Introduction

Polarizers, which control the polarization state of electromagnetic waves, have played a vital role as an essential element in a wide variety of applications such as polarimetry, imaging, switching, spectroscopy, and display^1,2^. However, conventional polarizers made of thin-film polymers or crystal films are vulnerable to high temperature and constant light illumination, which cause a significant performance degradation over time. Additionally, both polymer and crystal materials have strong absorption in the infrared (IR) spectral range, which limits their potential for IR applications. Wire-grid polarizers (WGPs), which consist of a periodic array of subwavelength metallic wires on a transparent substrate, have emerged as an attractive alternative to polymer polarizers because of their compactness, durability, broadband transmission, and wide-angle performance^1,3,4^. For transverse magnetic (TM) waves with an electric field oscillation direction perpendicular to the subwavelength wires of the WGPs, electron movement cannot be induced across the narrow width of the metal wires, thereby enabling the incident TM wave to pass through the WGPs. In contrast, when the oscillating direction of the electric field for a transverse electric (TE) wave is parallel to the wires of the WGPs, the electrons can freely move along the length of the metal wires. Hence, the WGPs for TE waves function as a typical metal, where the incident light is reflected. However, IR-transmitting substrates such as silicon (Si) and germanium (Ge) have high refractive indices that produce high reflection losses, which still restrict their high-performance use in the IR applications^3^. Although the transmission of TM waves could be improved by reducing the metal duty cycle of the WGPs, the polarization extinction ratio $({\rm{PER}}=\frac{{T}_{TM}}{{T}_{TE}})$, (*i.e*., the ratio of a parallel transmission (*T*_*TM*_) to a perpendicular transmission (*T*_*TE*_), which is also one of the most important characteristics of the WGPs, becomes poor as a result of the greater increase in (*T*_*TE*_)^1,3,5^.

The simplest approach to suppress high reflections from the surfaces of IR-transmitting substrates might be the use of a single layer with a low refractive index as an anti-reflective (AR) coating^6^. However, it is difficult to achieve broadband characteristics with a single AR layer. Although microstructures directly patterned at the subwavelength scale on a chalcogenide glass via an imprinting process have been demonstrated to mitigate the reflection losses, the PER was found to be less than 20 dB due to a reduction of the TM transmission at the short wavelength regime arising from the diffraction and a high leakage loss of the TE transmission resulting from the misalignment of the direction of the imprinted chalcogenide glass grating with that of the metal wire grating^7^. Besides, handling the WGP device with the microstructure-based AR surfaces is difficult. A zirconia (ZrO_2_) film based on a sol–gel technique was also demonstrated to improve the IR transmission^6^. However, a sol–gel-derived ZrO_2_ film contains micropores and nanopores that can generate scattering losses, and it would be challenging to use such a rough surface as a substrate in the fabrication of WGP devices. Moreover, the hydroxyl group in the film, which leads to large absorptions at mid-IR wavelengths, needs to be completely eliminated by a post annealing process at a very high temperature (*e.g*., 800 °C). Therefore, there is a critical need to develop a new strategy that can efficiently reduce the high reflection losses.

**Figure 1:**
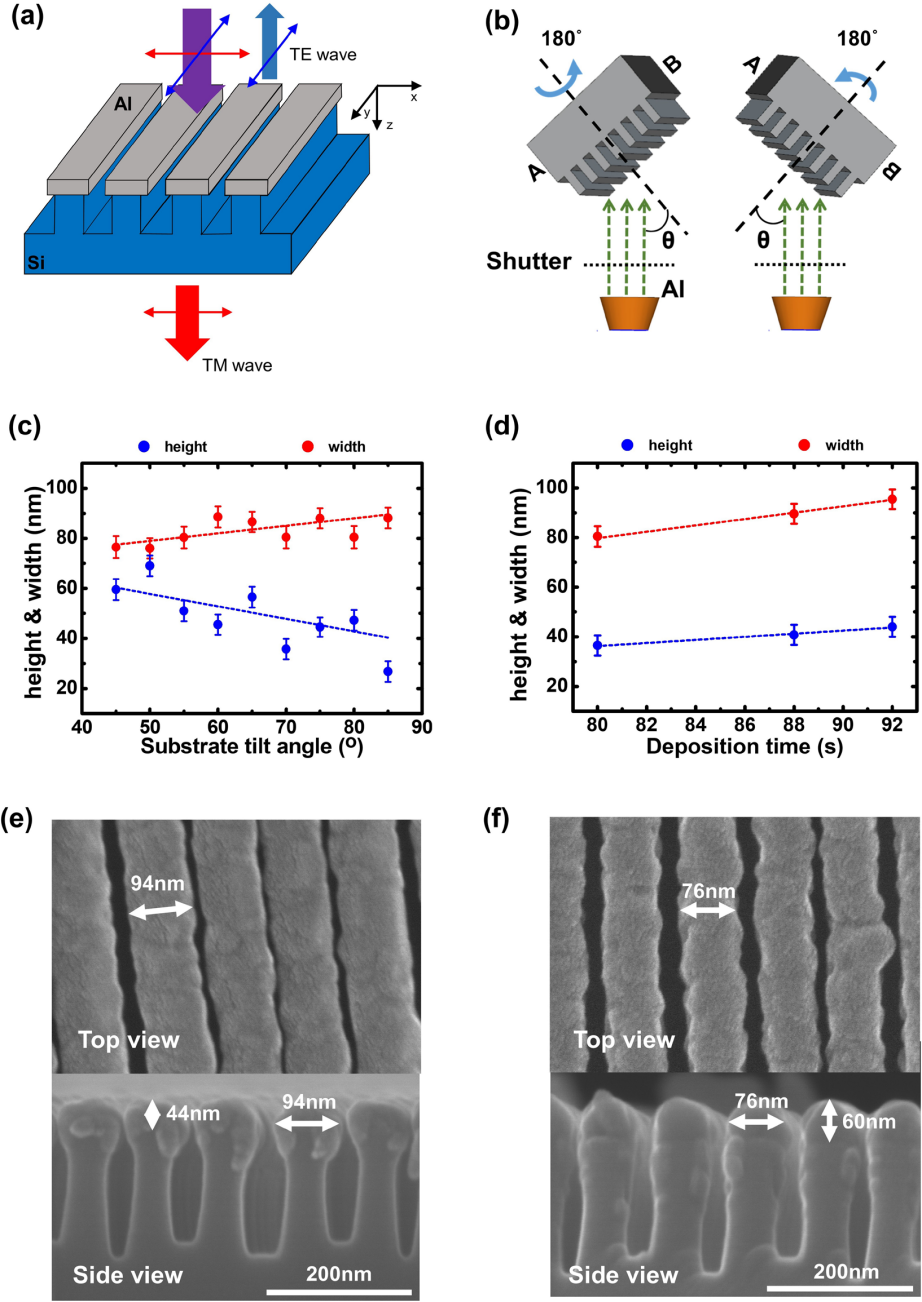
(**a**) Schematic view of proposed psWGP, [air|Al nanowires|Si nanopatterns|Si substrate]. (**b**) Schematic illustrations of a serial bi-deposition method of OAD. (**c**) and (**d**): The dependence of the Al nanowire width and height as functions of (**c**) a slanting substrate angle and (**d**) a deposition time. The Al deposition rate was 0.5 nm/s, and four serial bi-deposition turns were used. The width and the height, which are represented by colored circles, are determined by averaging values obtained from 16 positions in the SEM images. A deviation from the average value is about ±4 nm that is denoted by an error bar. The dashed lines are guidelines presenting a trend. (**e**,**f**): Top (top) and cross-sectional views (bottom) of SEM images of the fabricated psWGPs, where (**e**) the Al width is 94 nm and air gap is 6 nm; and (**f**) the Al width is 76 nm and air gap is 24 nm. These dimensions are the values averaged from 16 sites in the SEM images. Their OAD conditions include (**e**) a substrate angle of 70°, four turns, a deposition rate of 0.5 nm/s, and a deposition time of 92 s; and (**f**) a substrate angle of 45°, four turns, a deposition rate of 0.5 nm/s, and a deposition time of 80 s. The pitch of the Si nanopatterns is 100 nm, with a height of 140 nm and width of 50 nm.

In this study, we experimentally demonstrated highly efficient IR WGPs, [air|Al nanowires|Si nanopatterns|Si substrate], as shown in Fig. 1(a), where a thin aluminum (Al) layer was selectively deposited on the Si nanopatterns of a substrate using an oblique angle deposition (OAD) technique^8^. This new WGP structure is distinctly different from the conventional WGP structure [air|Al nanowires|Si substrate], where an array of subwavelength metallic nanowires is directly patterned on a flat substrate^9^. A high transmittance exceeding 80% was achieved over the entire mid-wavelength IR (MWIR) range of 3000–5000 nm. Additionally, the effects of the narrow air gap between the metallic nanowires on the optical properties of the MWIR WGPs were rigorously explored. Finally, the transmission enhancement of the proposed WGP structure for TM waves was examined by exploiting a combination of an effective medium approximation and an admittance diagram. The presented approach could offer insights for the future design of the MWIR WGPs with high efficiency, thereby potentially extending the range of possible IR applications.

## Results and Discussion

Figure 1(a) shows a schematic diagram of the proposed WGP structure with the selective deposition of Al nanowires on the patterned Si substrate, which had a period of 100 nm, height of 140 nm, and width of 50 nm. In the following, we refer to the proposed WGP [air|Al nanowires|Si nanopatterns|Si substrate] as psWGP, where ps refers to the patterned substrate. Transverse electric (TE) polarized light waves, where the oscillation direction of the electric field on the y-axis was aligned parallel in the direction of the metal nanowires (blue), were reflected, whereas transverse magnetic (TM) polarized light waves with an electric field whose oscillating direction in the xz-plane was perpendicular to the metal gratings (red) were able to pass through the WGP structure. Figure 1(b) presents schematic illustrations of a serial bi-deposition method, where a substrate tilted at an oblique angle of *θ* with respect to the substrate normal is rotated by a half-cycle (180°) every turn to selectively deposit Al only on top of the Si nanopatterns in the Si substrate and obtain the desired height (*h*) and width (*w*) for the Al nanowires. Figure 1(c) gives the dependence of the width (red) and height (blue) of the Al nanowires on the slanting angle of the substrate with four half-cycle turns at the fixed deposition rate of 0.5 nm/s, as measured by a quartz crystal monitor. When the substrate tilt angle is increased, the width of the Al nanowire increases, while the height decreases. Figure 1(d) provides the relationship between the deposition time and width (height) of the nanowires at the fixed substrate tilt angle of 70° and fixed deposition rate of 0.5 nm/s, showing that both the width and height of the Al nanowires increase with the deposition time. As shown in Figures 1(c,d), the dimension of the Al nanowires can be readily controlled by adjusting the substrate tilt angle and deposition time. Figures 1(e,f) display the top views (top) and cross-sectional views (bottom) of scanning electron microscopy (SEM) images of fabricated WGP samples with different deposition conditions for the serial bi-deposition method. They clearly show that Al nanowires are selectively placed on top of the Si nanopatterns under the OAD conditions: (e) substrate tilt angle of 70°, four half-cycle rotations, deposition rate of 0.5 nm/s, and deposition time of 92 s; and (f) substrate tilt angle of 45°, four half-cycle rotations, deposition rate of 0.5 nm/s, and deposition time of 80 s. The corresponding widths and heights of the Al nanowires are estimated to be 94 and 44 nm for (e), and 76 and 60 nm for (f), respectively. 16 spots in the SEM images were chosen to measure the width, height, and gap between the Al nanowires. 94 nm (width) and 44 nm (height) in Fig. 1(e), and 76 nm (width) and 60 nm (height) in Fig. 1(f) are values averaged from the measured dimensions in 16 spots. A deviation from the averaged value is found to be ±4 nm, which is indicated by an error bar in Fig. 1(c,d).

**Figure 2:**
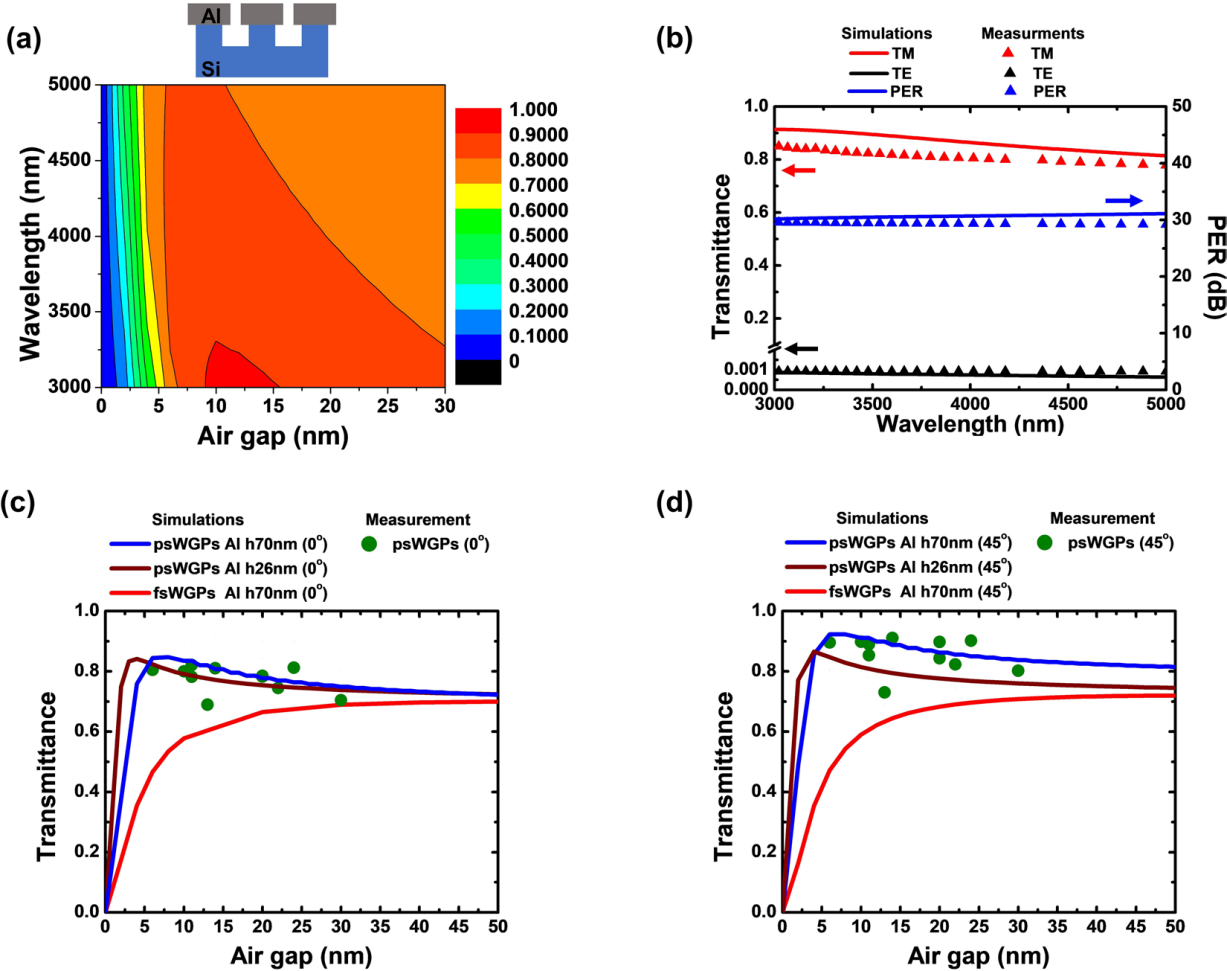
(**a**) 2D contour map of TM transmittance of psWGP, [air|Al nanowires|Si nanopatterns|Si substrate] as functions of air gap between Al nanowires and wavelength, as calculated using FDTD method. The height of the Al nanowires is 70 nm, and the period is 100 nm. (**b**) Measured and simulated transmission spectra (red for TM wave, black for TE wave), and polarization extinction ratio (blue) of the fabricated psWGPs with an air gap of 6 nm in the MWIR range. Measured and simulated TM transmittances at a fixed wavelength of 4000 nm (**c**) at normal incidence and (**d**) at 45° as a function of the air gap. Simulation data (red for fsWGPs of [air|Al nanowires|Si nanopatterns|Si substrate], blue and brown for psWGPs with Al heights of 70 and 26 nm, respectively) were obtained using the FDTD method. Experimental data (green circles) were obtained using FTIR.

A 2D contour plot of the TM-wave transmittance calculated by FDTD software for the proposed WGP structure is provided as a function of the air gap and wavelength in Fig. 2(a), where a 70-nm-thick Al nanowire is deposited only on top of the Si nanopatterns with a 100-nm period. It shows that the overall transmittance for the TM polarization in the MWIR wavelength regime of more than 85% increases with a decrease in the air gap between the Al nanowires, with the peak transmittance achieved with an air gap of 6 nm. We note that a surface reflection of ~30% that occurs at the bottom side of the Si substrate is not considered, because this can be improved using the previously reported AR approaches^5–7^. Figure S1 in the Supplementary Information (SI) presents one exemplary AR coating comprising 6 dielectric layers, which can highly suppress the reflections over the MWIR range of 3000–5000 nm, and the transmission spectra of a bare Si substrate with and without the designed AR coating. The transmission enhancement for the TM wave in such a narrow air gap has not been observed with the conventional WGP structures in the previous reports (Fig. S2 (a) in SI)^1,9^. In the following, we refer to the conventional WGPs, [air|Al nanowires|Si substrate], as fsWGPs, where fs refers to a flat substrate. Figure 2(b) shows the measured (triangles) and calculated (solid lines) transmission spectra of the psWGP structure at a fixed air gap of 6 nm (Al height: 44 nm) for TM (red) and TE polarizations (black), where the measured profiles are a good match for the calculated results. It shows that the measured transmittance of the psWGP with a 6-nm air gap is higher than 80% over the entire MWIR range of 3000–5000 nm, whereas the calculated transmittance of the fsWGP structure with an air gap of 6-nm drops to 46.0% (Fig. S2 (b) in SI). The polarization extinction ratio $(\,{\rm{PER}}=\frac{{T}_{TM}}{{T}_{TE}})$ of the psWGP structure is estimated from the measured transmittance and included in Fig. 2(b) (blue). The average PER in the MWIR region of 3000–5000 nm is found to be approximately 29 dB, which is much better than the PER values reported in previous studies^6^. The simulated transmission spectra generally show good agreement with the measured profiles with the differences being that the experimental results present relatively lower values of the transmittance than the simulated spectra. This could be ascribed to a non-consistent nanowire gap and a line edge roughness of the fabricated psWGP samples. The effect of the air gap on the transmittance is clearly observed in the transmittance at 4000 nm, as shown in Figures 2(c,d). Figure 2(c) displays that the transmittances of the psWGPs at normal incidence (blue and brown curves correspond to psWGPs with Al heights of 70 and 26 nm, respectively) become higher than those of the fsWGPs (red solid line) for air gaps ranging of 0-50 nm. The measured transmittances of the psWGPs with various air gaps under the different deposition conditions at normal incidence (green dot), which were obtained using Fourier-transform infrared spectroscopy (FTIR), are plotted together with the calculated curves in Fig. 2(c), where most of the measured data are located between two simulated curves of the psWGPs with the Al height of 70 nm and 26 nm. The same curves obtained at 45° are given in Fig. 2(d), all of which present relatively higher transmittances compared to those at normal incidence. As seen from these figures, a similar trend of an increasing transmittance with a decreasing air gap until 6 nm is observed. As noticed from the cross-sectional SEM images shown in Figures 1(e,f), the actual shape of the Al nanowires deposited using the OAD method for the fabricated psWGP structure is not a rectangle, but an ellipsoid. This is because of the inherent characteristics of a thin film growth using the OAD method. Although the psWGP structure looks like a mushroom in practice, it has been found that the difference between the TM transmittances attained from the ellipsoidal and rectangular models is quite negligible (Fig. S3 in SI). We also note that although the spacing between the Al nanowires was not completely regular as shown in Figures 1(e,f), it was confirmed that such irregularities for the Al nanowires, which arise during the stitching process of the spacer patterning technique used to fabricate the Si nanopatterned substrate, had a fairly insignificant effect on the optical performance of the psWGP (Fig. S4 in SI). Therefore, all the simulations of the psWGP structure were performed based on the rectangular model with a period of 100 nm and a linewidth of 50 nm in this work.

**Figure 3:**
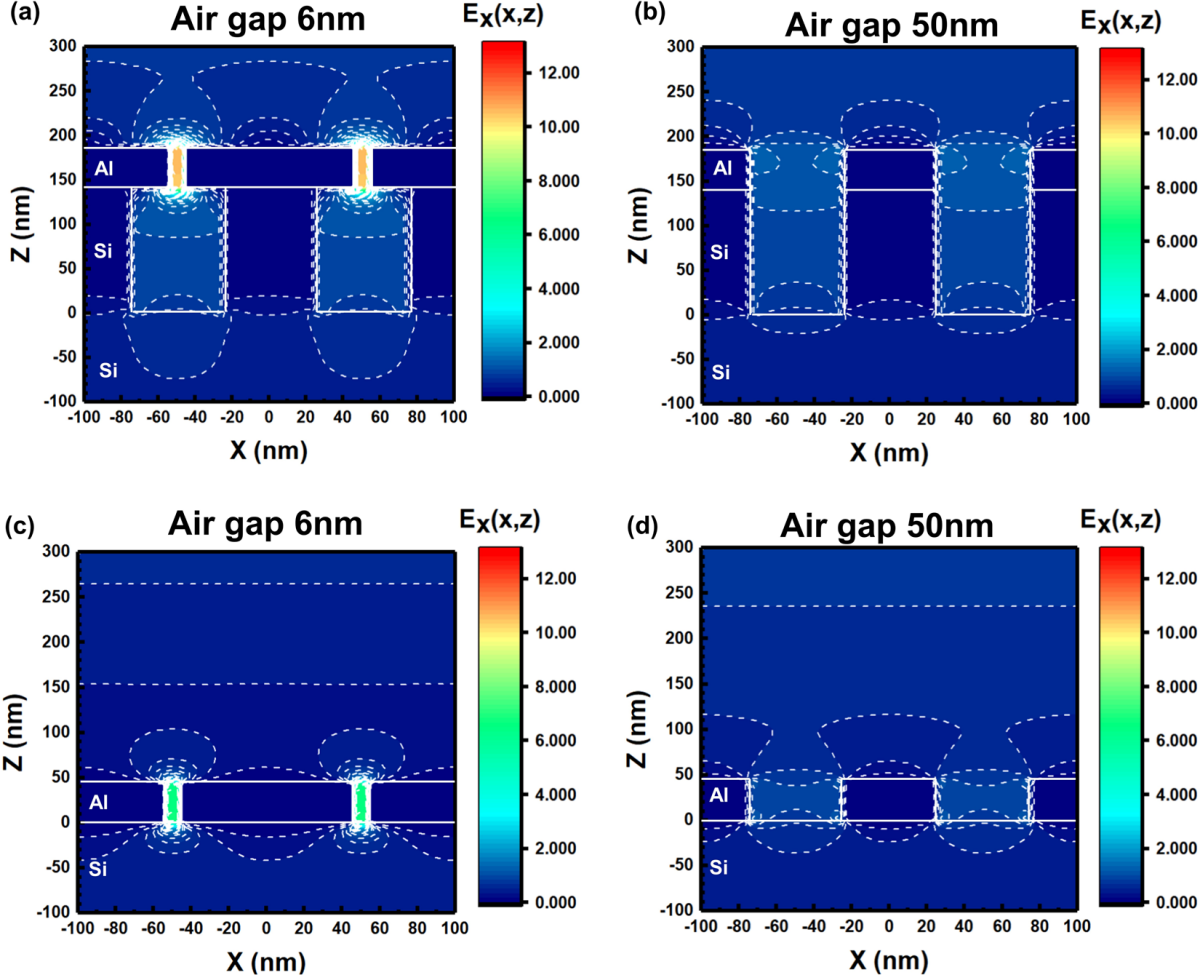
Electric field distributions of psWGPs [air|Al nanowires|Si nanopatterns|Si substrate] with air gaps of (**a**) 6 nm and (**b**) 50 nm. FDTD method was used. (**c**) Electric field profiles of fsWGPs [air|Al nanowires|Si substrate] with air gaps of (**c**) 6 nm and (**d**) 50 nm.

As previously observed, the transmittance of the psWGP increased with a decrease in the air gap of the Al nanowires, which was completely different from the fsWGP. To explore how the air gap affected the optical property of the psWGP, the electric field distributions of device structures with different air gaps were studied using FDTD. The electric field profiles of psWGP structures (Figures 3(a,b)) and fsWGP structures (Figures 3(c,d)) with air gaps of 6 and 50 nm at a fixed Al height of 44 nm and period of 100 nm at 4000 nm are provided in Fig. 3. The patterned Si substrate has a period of 100 nm, height of 140 nm, and width of 50 nm. As can be seen from the figures, the 6-nm air gap of the psWGP in Fig. 3(a) shows a higher electric field than the 50-nm air gap in Fig. 3(b). A similar behavior is observed from the fsWGP in Figures 3(c,d). However, it is noted that the psWGP structure with the 6-nm air gap in Fig. 3(a) presents an enhanced electric field compared to the fsWGP with the 6-nm air gap in Fig. 3(c). Such strong electric field enhancements in the narrow air gap are responsible for the transmittance enhancements of the psWGPs. These are also attributed to the better admittance matching, which will be investigated later. A similar transmittance enhancement from the metallic patterns for the TM wave in a terahertz (THz) wavelength regime was also observed, which can be ascribed to the high electric field in the narrow air gap^10^. The Poynting vector distributions of the psWGP structures with different air gaps can be found in Fig. S5 in SI.

**Figure 4:**
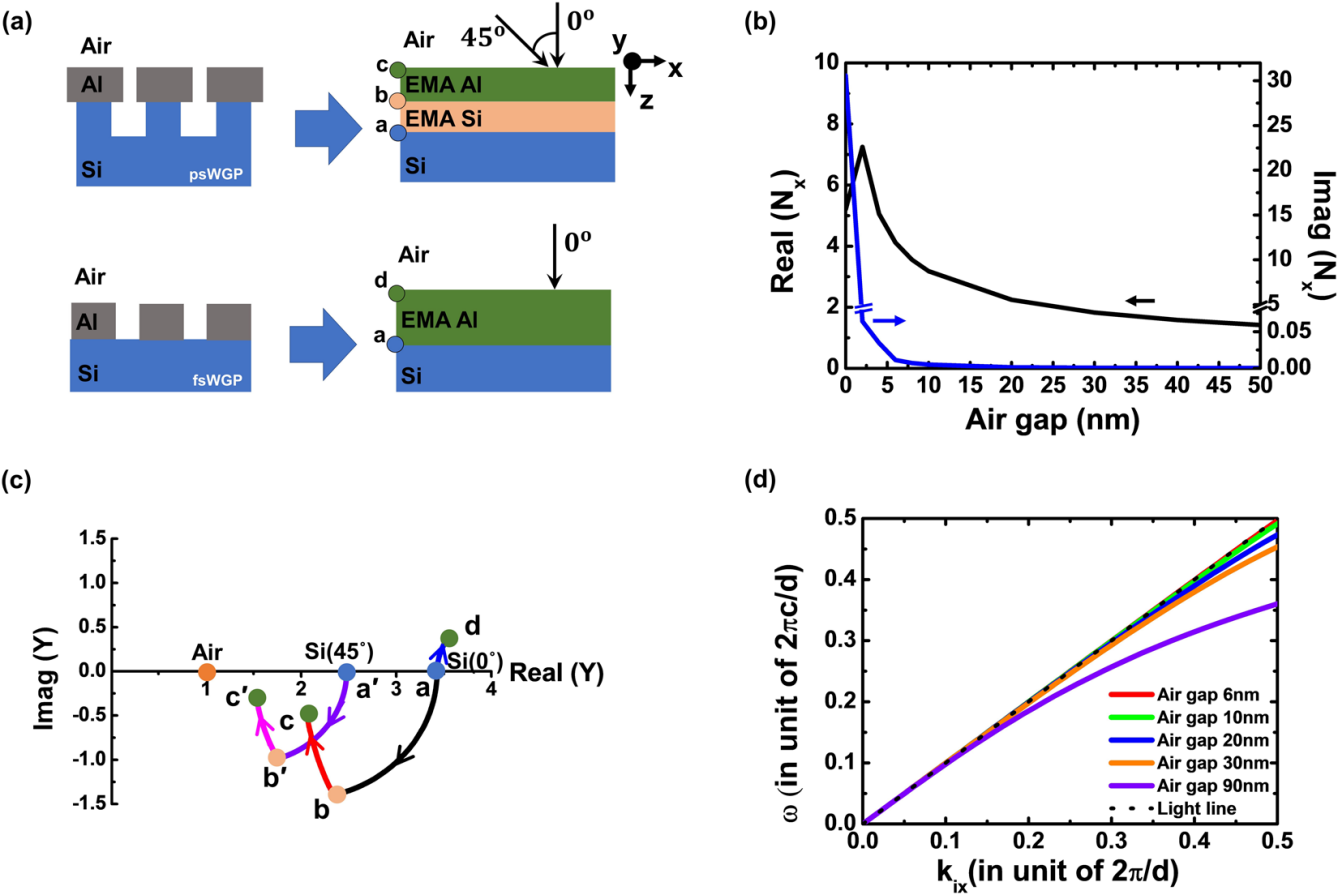
(**a**) Schematic illustrations of the psWGP(top) and the fsWGP (bottom) before (left) and after (right) applying EMA method. (**b**) Complex refractive index (*N*_*x*_) of the Al WGP for TM wave at 4000-nm wavelength as a function of the air gap between nanowires. (**c**) Admittance diagram showing the loci for the psWGP of [air|EMA Al nanowires|EMA Si nanopatterns|Si substrate] and the fsWGP of [air|EMA Al nanowires|Si substrate]. At the normal TM wave incidence, the admittance of the [air|EMA Al nanowires|EMA Si nanopatterns|Si substrate] starts from a, arrives at b, and ends at c, while the modified admittance at the 45° incidence angle follows a′, b′, and c′. The admittance of the fsWGP of [air|EMA Al nanowires|Si substrate] at the normal TM wave incidence starts from a and ends at d. Both the Al nanowires with the 6-nm air gap and the nanopatterned Si substrate with the 50-nm air gap are modeled as anisotropic uniaxial thin films by the EMA at the 4000-nm wavelength. The EMA Al-nanowire layer thickness is 44 nm, and the EMA Si-nanopattern layer thickness is 140 nm. (**d**) The dispersion characteristics of the Al WGP is presented as a function of the incident wave vector in the x direction. In the case of the Al WGP with the air gap of 6 nm explored in this paper, the dispersion curve is very close to the light line, suggesting that the TM wave can even pass through a narrow air gap with high transmittance.

To further investigate the improved optical property of the psWGP structure, a surface admittance analysis was performed using the effective medium approximation (EMA) method, in which an array of Al nanowires was approximated as an anisotropic uniaxial thin film. The EMA is a method that is used to describe the macroscopic properties of periodic nanostructures in the long wavelength limit, in which the period of the nanowire is 1/40 or less of the incident wavelength^11,12^. The period of the WGP structure studied in this work was 100 nm, which was less than the wavelengths in the entire MWIR range of 3000–5000 nm. Because of the one-dimensional (1D) structure configuration, anisotropic uniaxial thin films with different refractive indices for perpendicular (TM) and parallel (TE) polarizations were used^13^. Figure 4(a) depicts schematic diagrams of the psWGP (top) and the fsWGP (bottom) structure before and after applying the EMA method, the latter of which shows that the 1D array of Al nanowires is modeled as a uniaxial film. The permittivity of a uniaxial film with an optical axis in the x direction for TM and TE polarizations can be expressed as follows^3^:

1 $$\frac{1}{{\varepsilon }_{x}}=\frac{f}{{\varepsilon }_{m}}+\frac{1-f}{{\varepsilon }_{a}},\,for\,TM\,wave$$

2 $${\varepsilon }_{y}={\varepsilon }_{m}\,f+{\varepsilon }_{a}(1-f),\,for\,TE\,wave,$$

where *ε*_*m*_ is the dielectric constant of the metal, ε_a_ is the dielectric constant of the air, and *f* is the fill factor of Al in a period that corresponds to the Al duty-cycle^13^. The complex refractive index $({N}_{x}=\sqrt{{\varepsilon }_{x}})$ of the array of the Al nanowires calculated from the dielectric constant (*ε*_*x*_) at the 4000-nm wavelength as a function of the air gap is provided for the TM polarized light wave in Fig. 4(b). Decreasing the air gap from 50 to 5 nm leads to an increase in Re(*N*_*x*_) from 2 to 5, with Im(*N*_*x*_) ≈0, indicating that the Al nanowire structure behaves as a dielectric material with high transmittance for the TM wave. If the air gap is less than 5 nm, Re(*N*_*x*_) decreases and Im(*N*_*x*_) increases. The calculated complex refractive index (N_y_) for the TE polarization is provided in Fig. S6 in SI, in which Im(N_y_) is much larger than Re(N_y_), suggesting that the Al nanowire structure behaves as a metal with high reflectance for the TE wave. The wavelength-dependence of *N*_*x*_ and N_y_ at an air gap of 6 nm between the Al nanowires presented in Fig. S7 (a) shows very small variation, indicating the broadband transmission characteristic of the TM waves in the MWIR region. The complex refractive index of the Si nanopatterns with an air gap of 50 nm are is presented in Fig. S7 (b), indicating the dielectric behavior in the MWIR region.

After the anisotropic refractive index of each layer of the WGP structure was obtained using the EMA method, the optical properties of the psWGP structure, i.e., the [air|EMA Al-nanowires|EMA Si-nanopatterns|Si substrate], were investigated using the transfer matrix and admittance analysis. The transfer matrix of a uniaxial layer for the TM wave can be given by the following:

3 $$[M]=[\begin{array}{cc}\cos \,\delta  & i\frac{\sin \,\delta }{\eta }\\ i\eta \,\sin \,\delta  & \cos \,\delta \end{array}],$$

where the optical phase thickness is δ = *k*_*z*_*h*, the modified admittance of the uniaxial film with N_y_ = N_z_ is $\eta ={\varepsilon }_{x}\frac{{k}_{0}\cos \,{\theta }_{i}}{{k}_{z}}={N}_{x}{N}_{z}\frac{\cos \,{\theta }_{i}}{\sqrt{{N}_{z}^{2}-{S}^{2}}}$, Snell’s law is ${S}={n}_{i}\,\sin \,{\theta }_{i}={N}_{z}\,\sin \,{\theta }_{f}={n}_{sub}\,\sin \,{\theta }_{sub}$, the thickness is *h*, and the dispersion relation is ${k}_{0}^{2}=\frac{{k}_{x}^{2}}{{\varepsilon }_{z}}+\frac{{k}_{z}^{2}}{{\varepsilon }_{x}}$, in which *k*_*x*_ and *k*_*z*_ are propagation vectors in the *x* and *z* directions, respectively. The optical phase thickness can be rewritten as $\delta =\frac{2\pi }{\lambda }{N}_{x}h\sqrt{1-\frac{{S}^{2}}{{N}_{z}^{2}}}=\frac{2\pi }{\lambda }{N}_{x}h\,\cos \,{\theta }_{f}$^14^. Then, the surface admittance *Y* of the thin film is given by

4 $$Y=\frac{C}{B},$$

where the normalized electric (*B*) and magnetic (*C*) fields are obtained by the multiplication of two transfer matrices of uniaxial layers,

5 $$[\begin{array}{c}B\\ C\end{array}]=[{M}_{EMAAl}][{M}_{EMASi}][\begin{array}{c}1\\ {Y}_{sub}\end{array}],$$

where ${Y}_{sub}={n}_{sub}\frac{\cos \,{\theta }_{i}}{\cos \,{\theta }_{sub}}$ is the modified substrate admittance. Then, the reflectance is given by

6 $$R={|\frac{{Y}_{i}-Y}{{Y}_{i}+Y}|}^{2},$$

where *Y*_*i*_ = n_i_ is the admittance of the incident medium^15^. Figure S8 in SI displays the spectral transmittance curves of psWGP for TM waves at normal and 45° incident angles, as calculated using the EMA and FDTD methods. It shows that the *T*_*TM*_ calculated by the EMA method is in good agreement with that obtained by the FDTD method.

The admittance diagram traces the locus of surface admittance *Y* of the thin films on the complex plane as the optical thickness of the thin film is increased. It is widely used to analyze the reflectance, transmittance, phase change, etc. according to the optical thickness of the thin film^15,16^. It can be noted from Eq. (6) that the minimized distance between the admittance of the incident medium and the termination point of the admittance at the top surface of the film corresponds to a lower reflectance, and thus a higher transmittance^15^.

Figure 4(c) shows the trajectories of the optical admittances of both the psWGP and fsWGP structures at 4000 nm with an air gap of 6 nm. The admittance of the fsWGP [air|Al-nanowires |Si substrate] begins with the Si substrate admittance “a” (3.42, 0) and ends at the surface admittance of the EMA Al nanowire layer “d” (3.52, 0.34), giving rise to the transmittance of *T*_*TM*_ = 68.4%. Such a low transmittance is attributed to the large distance from the final surface admittance point “d” to the air, which indicates that the high refractive index of the Si substrate limits the transmittance of the TM wave. On the other hand, for the proposed psWGP of [air|Al-nanowires|Si-nanopatterns|Si substrate], the starting admittance is that of the Si substrate “a” (3.42, 0), and the subsequent loci are determined by the following two layers, where the routes of the admittances traced out by the 140-nm-thick EMA Si-pattern layer and 44-nm-thick EMA Al-nanowire layer with a 6-nm air gap terminate at points “b” (2.34, −1.40) and “c” (2.06, −0.49), respectively. The final surface admittance point “c” at the interface between air and the EMA Al-nanowire layer is pretty close to the air admittance (1,0). Therefore, the much improved transmittance of *T*_*TM*_ = 85.5% is attained, as compared to that of the fsWGP (*i.e*., *T*_*TM*_ = 68.4%). This result is consistent with that in Fig. 2(c), in which the *T*_*TM*_ of psWGP is higher than that of fsWGP in a narrow air gap of less than 10 nm. Figure 4(c) also shows the trajectory of the modified admittances of the psWGP with an air gap of 6 nm at an oblique incident angle of 45°. The modified admittance starts from “a′” (2.47, 0) for the Si substrate and ends at point “c′” (1.54, −0.47), which is closer to the air admittance (1,0) than that at the normal incidence, resulting in a higher transmittance of *T*_*TM*_ = 92.3% compared to that at the normal incidence. This is consistent with that in Fig. 2(d), in which the *T*_*TM*_ of psWGP at 45° is higher than that at the normal incidence. The detailed calculation of each admittance point is provided in SI (section S6).

The anisotropic refractive index of WGP in the long wavelength limit obtained from the EMA method can be used to derive the dispersion characteristics of the WGP as follows:

7 $$\tan (\frac{\omega h}{2c})={\varepsilon }_{x}\frac{\sqrt{{k}_{ix}^{2}-{(\frac{\omega }{c})}^{2}}}{\frac{\omega }{c}},$$

by assuming that the amplitude transmission coefficient of [air|EMA Al-nanowires|air] for the TM wave approaches infinity, and *k*_*ix*_ is the incident wave vector in the x direction^17–19^. In the MWIR regime, Al is close to the perfect electric conductor, because the |*ε*_*m*_| of Al is very large (~1000). Then, from Eq. (1), the permittivity ε_*x*_ of a uniaxial [EMA Al-nanowire] layer can be approximated as ${\varepsilon }_{x}=\frac{d}{a}$, where *d* is the period of the Al nanowires, and *a* is the air gap. This suggests that the Al-nanowire WGP behaves as a dielectric film with a refractive index of ${N}_{x}=\sqrt{\frac{d}{a}}$ for the TM wave, and the dispersion relation of the WGP can be represented using the geometrical parameters of the period, air gap, and height of the nanowires. It is apparent that the dispersion curve exhibited in Fig. 4(d) becomes closer to the light line with the decreasing air gap, implying that the fundamental waveguide mode for TM waves passes through the narrow air gap with an effective index of 1. Details on the dispersion relation of the WGP in the long wavelength limit are provided in SI (section S7).

**Figure 5:**
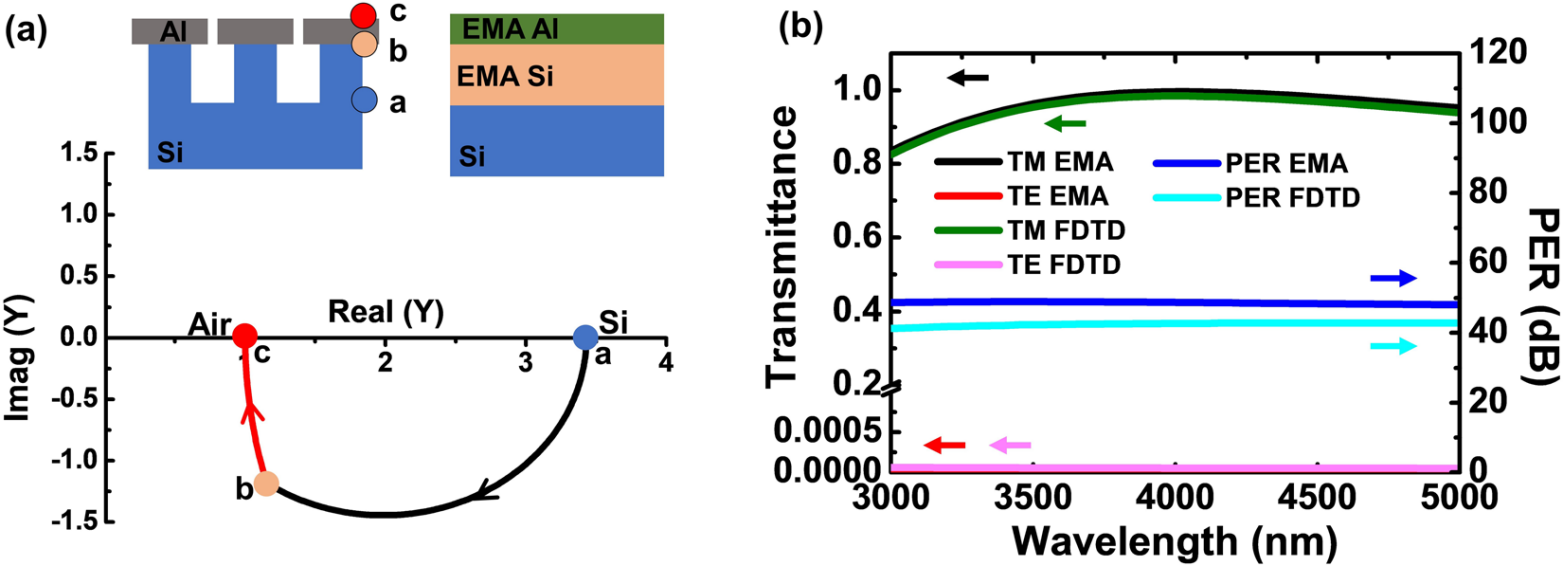
(**a**) Schematic representations and an admittance diagram of the optimized psWGP design with perfect transmittance at 4000 nm: [air|Al nanowires (*h* = 80 nm, *w* = 90 nm, air gap = 10 nm)|Si nanopatterns (*h* = 305 nm, *w* = 50 nm, air gap = 50 nm)|Si substrate]. (**b**) Transmission spectra at normal incidence for TM and TE polarizations, and calculated PER of the optimized psWGP structure studied in (**a**), calculated by using both the EMA and the FDTD methods.

If the termination point of the surface admittance in the admittance diagram can be designed to be equal to the air admittance by altering the dimension of the WGP device structure, i.e., a perfect admittance matching condition, we could achieve a reflection of 0%, and thus almost 100% transmittance for the TM wave. Note that both Si and Al have negligible absorptions in the MWIR range. Figure 5(a) shows schematic views of the psWGP whose dimensions were designed to achieve the perfect admittance matching condition, along with their admittance diagrams. To accomplish the perfect admittance matching for the psWGP structure, the height and width of the Al nanowires were designed to be 80 and 90 nm, respectively, on top of the Si nanopatterns with a height of 305 nm at a fixed period of 100 nm, leading to anisotropic refractive indices of *N*_*x, Al*_ = 3.176−*i*0.005 and N_x, __*Si*_ = 1.358 for the EMA Al nanowire layer and EMA Si nanopattern layer, respectively, as exhibited in Fig. 5(a). In the admittance diagram of Fig. 5(a), the trajectory represented by the black solid line arises from the 305-nm-thick Si nanopattern layer, and the remaining locus describes the perfect admittance matching with the 80-nm-thick Al nanowire film. Figure 5(b) presents TM and TE transmission spectra and PER values of the corresponding optimized WGP structure at normal incidence, which were calculated by using the EMA and the FDTD methods. As shown in the figure, almost 100% transmittance at 4000 nm, and > 96% average transmittance from 3000 nm to 5000 nm for the TM polarized light wave are achieved for the optimized psWGP design. The PER is found to be 48.6 dB, which is dependent upon the width and the height of the Al nanowires. It is important to note that the optimized psWGP design shown here is just one of the potential designs to achieve the perfect admittance matching.

## Conclusion

We have demonstrated an improvement in the transmission of TM waves in the narrow air gap of MWIR WGPs on a patterned substrate. The transmission of TM waves in the MWIR regime could be enhanced by decreasing the air nanogap between the Al nanowires of the psWGP on the nanopatterned Si substrate. This was ascribed to the fact that the electric field is very strong in a narrower air gap, and the nanopatterned Si layer acts as a matching layer, which leads to better admittance matching than the conventional WGP geometry that is typically fabricated on a flat substrate. Moreover, the optimized WGP design has been described, where the admittance can be perfectly matched by modifying the dimension of the WGP structure to achieve 100% transmittance for the TM wave. The strategy presented here could suggest a design principle for future IR WGPs with improved performance, thus making them more versatile for various applications, including polarimetry, target tracking, spectroscopy, and recognition.

## Methods

### Oblique angle deposition (OAD)

Al nanowires were selectively deposited on top of a nanopatterned Si substrate using the OAD method in an electron-beam-evaporation high-vacuum chamber, as illustrated in Fig. 1(b), where the substrate tilting angle (*θ*), number of half-cycle rotations, and total deposition time could be controlled to obtain the desired height (*h*) and width (*w*) for the nanowires. Nanopatterned Si substrates (3 cm × 3 cm) with a period of 100 nm, height of 140 nm, and width of 50 nm were procured from the National Nanofab Center in South Korea. When using the OAD method, the vapor of the evaporation material is incident upon the substrate at a predetermined angle when the material is deposited. An inclined columnar structure can be formed on the substrate when the vapor of the evaporation material is inclined at an oblique incidence angle of *θ*. The inclined column continues to grow from the substrate to the top surface of the film, because the vapor of the evaporation material does not reach the substrate because of the shadow effect of the tilted columns and self-limited surface diffusion of the evaporated materials during the deposition process^20–22^. Various types of nanostructured films, such as tilted columns, zigzag columns, and chiral nanostructures, can be fabricated depending on the angle of the vapor flux and condition of the substrate rotation in OAD^22^. In this study, we fabricated Al nanowires on top of the Si nanopatterns of the substrate using OAD. A serial bi-deposition method was used to form a zigzag nanostructure, in which the patterned Si substrate was tilted at an oblique angle and then rotated by half a turn^23^.

### Numerical simulations and optical characterizations

Simulations based on a finite-difference time-domain (FDTD) method^24^ were carried out to investigate the optical properties and electric field distributions of the WGPs. Because the 100-nm period of the WGP was much smaller than the IR wavelength, an effective medium approximation (EMA) was used to estimate the anisotropic refractive indices of each layer of the WGP structures to explore the enhanced transmittance in the narrow air gap engineered by OAD, which was studied using an optical admittance diagram^15,16^. The optical transmittance of the WGP in the MWIR range was measured using Fourier-transform infrared spectroscopy (FTIR, Nicolet iS5), and polarizers (Moxtek 3–5 C) were utilized to produce polarized light in the FTIR instrument.

## Supplementary information

Supplementary Information Nanogap Engineering for Enhanced Transmission of Wire-Grid Polarizers in Mid-Wavelength Infrared Region
