# Supplementary material for: Nanogap Engineering for Enhanced Transmission of Wire Grid Polarizers in Mid-Wavelength Infrared Region
Source: Sci Rep. 2019 Mar 12;9:4201. doi: 10.1038/s41598-019-40614-6 (PMC6414691; doi:10.1038/s41598-019-40614-6)
Supplement: Supplementary file 1 — Supplementary Information Nanogap Engineering for Enhanced Transmission of Wire-Grid Polarizers in Mid-Wavelength Infrared Region [file 41598_2019_40614_MOESM1_ESM.pdf]

Supplementary Information

Nanogap Engineering for Enhanced Transmission of Wire-Grid Polarizers in Mid-Wavelength Infrared Region

Wonyoung Kim[1], Minsuk Kim[1], Tae Young Kim[1], Hyunjin Choi[2], Myung-Jong Jin[3], Kyu-Tae Lee[1], Minbaek Lee[1], and Chang Kwon Hwangbo[1,*]

[1]Department of Physics, Inha University, 100 Inha-ro, Michuhol-gu, Incheon 22212, Republic of Korea

[2]Agency for Defense Development, Daejeon, Republic of Korea

[3]Department of Chemistry and Chemical Engineering, Inha University, 100 Inha-ro, Michuhol-gu, Incheon 22212, Republic of Korea

*hwangbo@inha.ac.kr

S1. Six-layer anti-reflection (AR) coating

S2. FDTD calculation results of the fsWGP

S3. Comparison of an ellipsoidal model with a rectangular model of the psWGP

S4. Poynting vector distributions of the psWGP structures with different air gaps

S5. Additional EMA analysis results

S6. Detailed calculation of admittance trajectory example.

S7. Dispersion relation of the wire grid polarizer

S1. Six-layer anti-reflection (AR) coating

Figure S1 (a) shows the schematic diagram of an exemplary 6-layer anti-reflection (AR) coating consisting of YF_3 and ZnS with optimized thicknesses for eliminating the reflection occurring at a back surface. Transmission spectra of a bare Si substrate with (red) and without (black) the designed AR coating are provided in Figure S1 (b). The reflection without the AR coating from a one surface of the Si substrate would be about 30% (Figure S1 (b) black solid line), which can be greatly suppressed by the optimized AR coating (Figure S1 (b) red solid line). The AR coated Si substrate presents 99.6% of an average transmittance over a wide wavelength ranging from 3000 nm to 5000 nm. Any other AR approaches would also be suitable for the elimination of the reflection occurring at the bottom surface of the Si substrate.

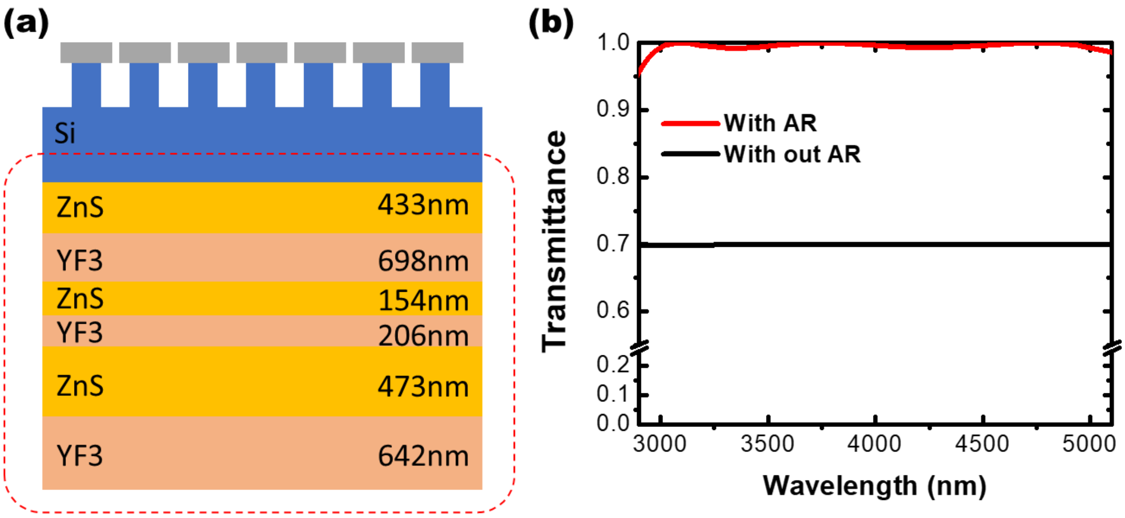

Figure S1. (a) Schematic diagram of an anti-reflection (AR) coating comprising 6 layers with optimized thicknesses to suppress the reflection from the bottom surface of the Si substrate. (b) Simulated transmission spectrum of the Si substrate with (red solid line) and without (black solid line) the AR coating.

S2. FDTD calculation results of the fsWGP

It has been found that the transmittance of the fsWGP structure ([air|Al nanowires|Si substrate]) for TM polarization decreases as the air gap decreases, which is different from what the psWGP structure ([air|Al nanowires|Si nanopatterns|Si substrate]) presents in Fig. 2(C). The psWGP structure with the air gap of 6 nm shows the average TM transmittance of 81% in the MWIR region, which is much higher than the average TM transmittance (46%) of the fsWGP structure with the same air gap (the surface reflection loss around 30% at the bottom part of the Si substrate is not considered). The TM transmittance of the fsWGP structure is pretty low over a wide wavelength range even with a large air gap of 30 nm, which indicates that anti-reflective (AR) coatings at the bottom of the Al nanowires are typically required to improve the transmittance of the conventional WGP structure (i.e., fsWGP) [RS1].

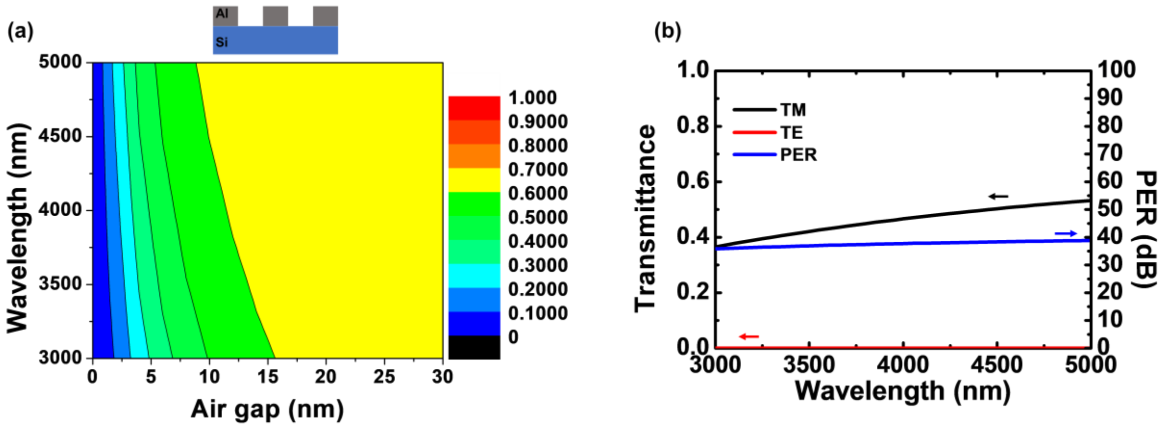

Figure S2. (a) 2D contour plot of a transmittance in the fsWGP structure as a function of an air gap and the wavelength, presenting that the transmittance decreases with decreasing the air gap of the fsWGPs. (b) Calculated transmission spectra of the fsWGP structure with the air gap of 6 nm for TM (black) and TE (red) polarizations, and its polarization extinction ratio (PER). The average TM transmittance of the fsWGP structure is 46.0% and the PER is 37.6dB in the MWIR region

S3. Comparison of an ellipsoidal model with a rectangular model of the psWGP

As can be seen from the cross-sectional SEM image in Fig. S3 (a), the cross-section of real Al-nanowires on top of Si-nanopatterns in the fabricated psWGP structure looks like a half-elliptic shape rather than a rectangle of ideal Al-nanowires and overfills in part the Si-nanopatterns. The half-elliptic shape that has been observed in other applications seems to be due to the intrinsic property of a thin film growth in the oblique angle deposition [RS2]. Although the psWGP structure looks like a mushroom in practice, it has been found that a difference between the TM transmittances attained from both the ellipsoidal and the rectangular models is quite negligible as shown in Fig. S3 (b), where the difference between the two curves is less than 1% (for the case of the psWGP with the air gap of 6 nm). Therefore, all the simulations in the main text were carried out by using the rectangular model for the simplicity.

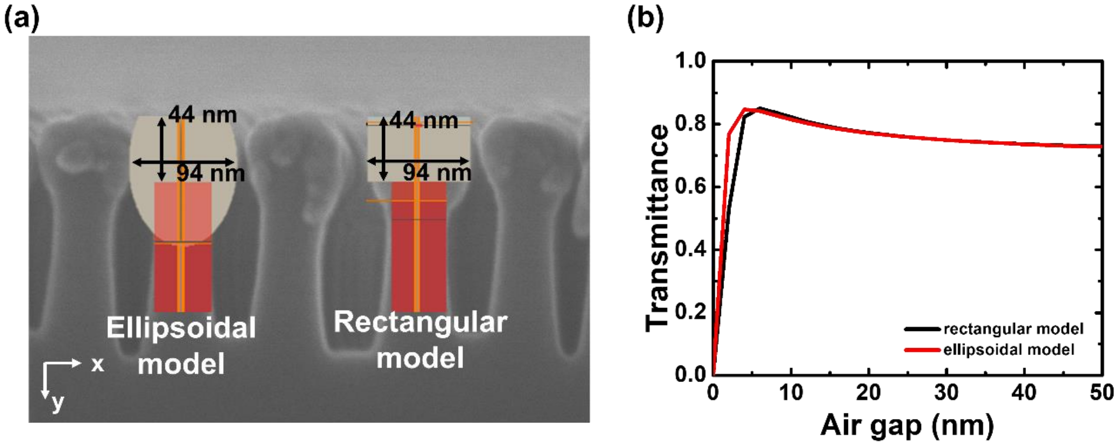

Figure S3. (a) Modeling of a real Al-nanowire with a round-shape (left, ellipsoidal model) and an ideal Al-nanowire with a rectangle (right, rectangular model) in a cross-sectional SEM image of the psWGP structure with the air gap of 6 nm. (b) TM transmittances of the psWGPs using the ellipsoidal (red) and the rectangular models (black) with different air gap at 4000 nm wavelength, calculated by using FDTD. Difference between the two curves is less than 1%.

SEM images of the psWGPs in Figures 1 (e) and (f) in the main text show that the air-gap between the nanowires is not equal in the top view of the real Al-nanowires, where one air-gap is wider than three. This originates from the Si-nanopatterns on a bare Si substrate. A line spacing of the Si-nanopatterns is wider than three. This could be happened in the stitching process of the spacer patterning technique of the Si-nanopatterns. As it has been found to be pretty difficult to fabricate the Si nanopatterns with a linewidth of 50 nm in a highly ordered manner over the large area, a top-down fabrication method based on stitchless stepper lithography and repetitive pattern downscaling technology was used for the

fabrication of the Si nanopatterned substrate. After preparing the Si nanopatterns with a period of 100 nm, the pattern pitch was further reduced by the initial nanopatterns exploiting the spacer patterning technology. Details on the fabrication technique can be found in a reference RS3.

Figures S4 (a) - (c) present schematic diagrams of the psWGP structures with the equal air gap (all air gaps are 6 nm, (a)), the unequal air gap (6 nm, 6 nm, 6 nm, 20 nm, (b)), and (6 nm, 8 nm, 7 nm, 20 nm, (c)). As is seen from the Figure 1 (e), a 20 nm gap exists and three nanopatterns whose spacing is close to 6 nm are repeated within a unit cell having a pitch of 400 nm, which is different from the structure dimension in the simulation where a 6 nm air gap and a 100 nm pitch are used. Figure S4 (d) describes calculated transmission spectra of the psWGP structures with all the air gaps are same at 6 nm and the pitch of 100 nm in a unit cell (black solid line), with the pitch of 400 nm and unequal air gaps of 6, 6, 6, and 20 nm in a unit cell (red solid line), and with the pitch of 400 nm and unequal air gaps of 6, 8, 7, and 20 nm in a unit cell (blue solid line) at normal incidence are displayed. Since a trivial discrepancy between the three curves, which is about 3% averaged from the MWIR range (3000 nm - 5000 nm), is observed, all the simulations with the period of 100 nm and the air gap of 6 nm were carried out to explore the optical properties of the psWGPs in the main text.

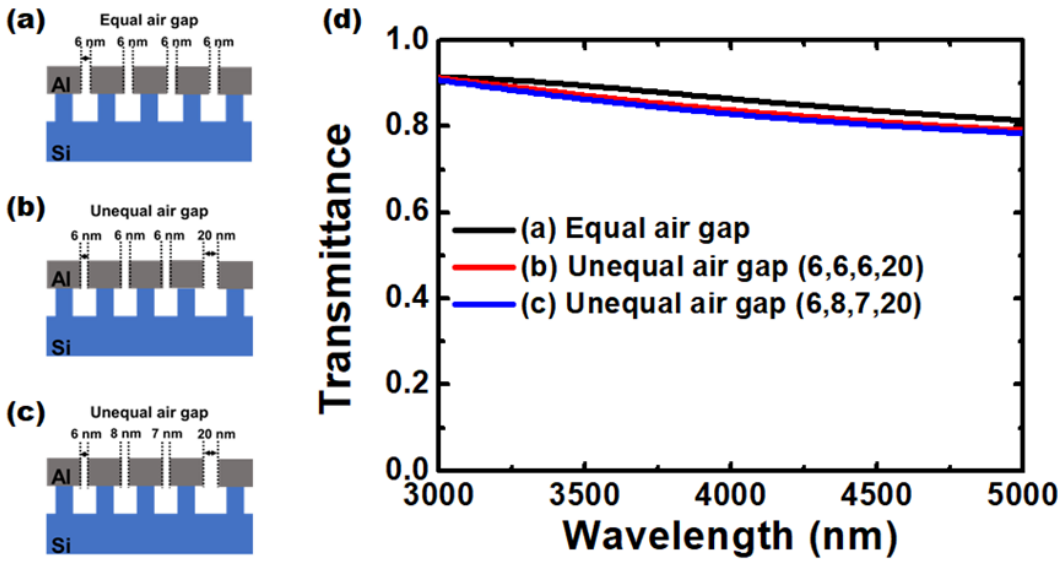

Figure S4. Schematic diagrams of the psWGP structures with (a) an equal air gap, (b) an unequal air gap (6 nm, 6 nm, 6 nm, 20 nm), and (c) an unequal air gap (6 nm, 8 nm, 7 nm, 20 nm). (d) TM transmission spectra of the psWGP structures with a pitch of 100 nm and an air gap of 6 nm in a unit cell (black), with a pitch of 100 nm and four different air gaps of 20, 6, 6, and 6 nm in a unit cell (red), and with a pitch of 100 nm and four different air gaps of 6, 8, 7, and 20 nm in a unit cell (blue), all of which were calculated by using FDTD.

S4. Poynting vector distributions of the psWGP with different air gaps

Figures S5 (a) and (b) display Poynting vector distributions of the psWGP structures with the air gap of 6 nm and 50 nm, respectively. As observed in the electric field profile analysis in the main text, the intensity of the Poynting vector becomes strong with decreasing the air gap, which corresponds to the enhanced TM transmittance of the psWGP in a narrow air gap.

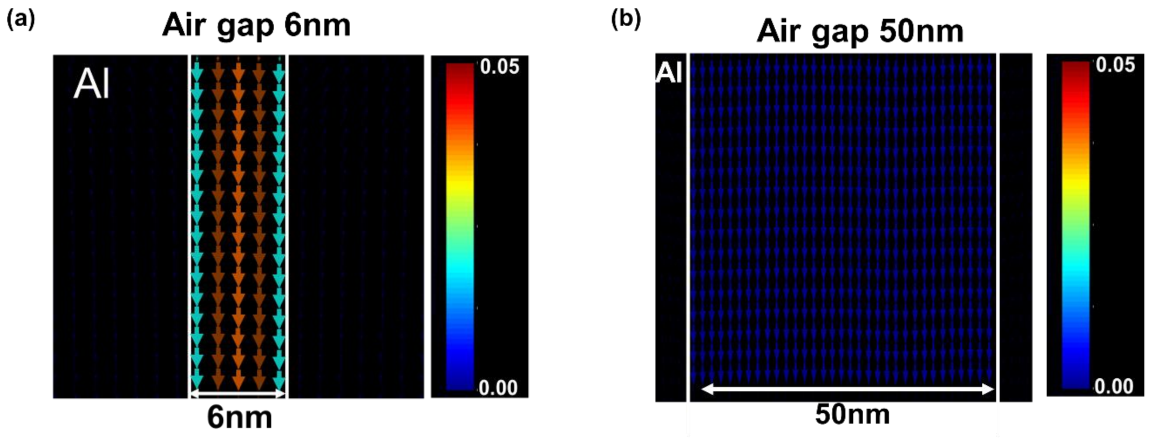

Figure S5. Poynting vector distributions of the psWGPs with the air gap of (a) 6 nm and (b) 50 nm.

S5. Additional EMA analysis results

N_x in Figure 2(b) of the main text, which corresponds to the complex refractive index of the anisotropic uniaxial Al-nanowires layer for TM polarization, shows a very small imaginary part, whereas N_z in Figure S5 of the SI that is the anisotropic uniaxial refractive index of the Al-nanowires for TE polarization shows a very high imaginary part as compared to a real part. This implies that the Al nanowires function as the dielectric (metal) for TM (TE) polarization, which can transmit (reflect) the incident light of TM (TE) polarization.

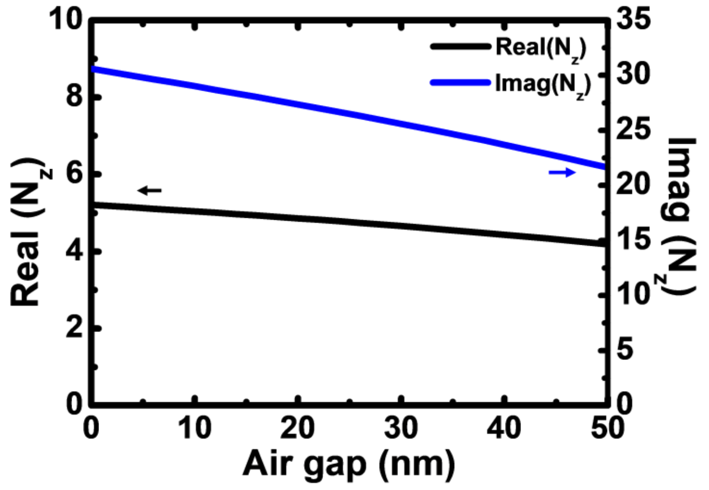

Figure S6. Complex refractive index (N_z) of an anisotropic uniaxial Al-nanowires layer for TE polarization at 4000 nm as a function of the air gap, calculated by using the EMA method.

In Figure S6, wavelength-dependent complex refractive indices of (a) the anisotropic uniaxial Al-nanowires layer with the 6-nm air gap and (b) the Si nanopatterns with the 50-nm air gap for both TM (N_x) and TE (N_z) polarizations are presented, in which the EMA method is employed for calculation. It is apparent that the anisotropic uniaxial refractive indices of both the Al nanowires and the Si nanopatterns are nearly insensitive with respect to the wavelength ranging from 3000 nm to 5000 nm, showing a negligible dispersion in the MWIR region.

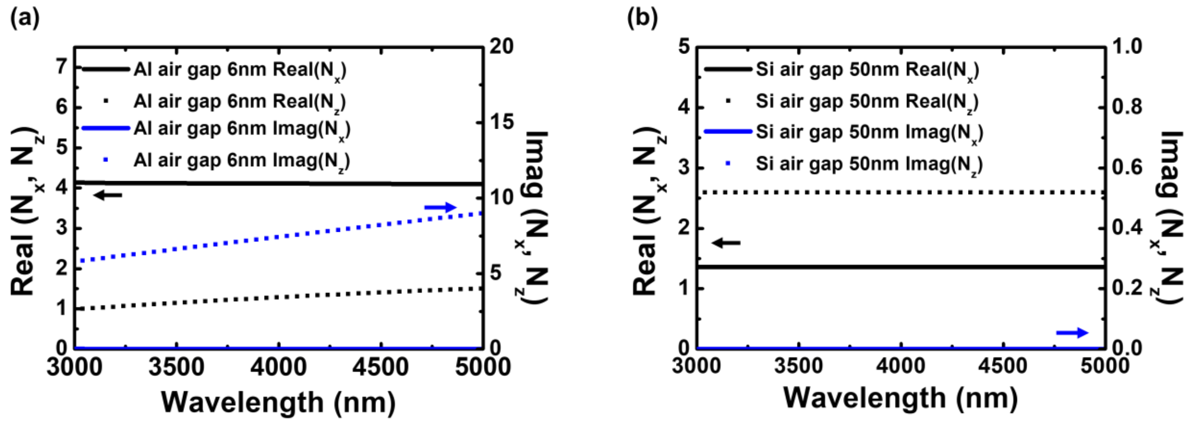

Figure S7. Calculated wavelength-dependent anisotropic uniaxial refractive indices of (a) the Al nanowires with the 6-nm air gap and (b) the Si nanopatterns with the 50-nm air gap using the EMA method, where N_x (N_z) corresponds to the TM (TE) wave.

Simulation results of TM transmittances of the psWGP structure with 70-nm Al height obtained from FDTD (black) and EMA (red) methods, along with some measured data points, at normal incidence (0˚) and oblique incidence (45˚) are provided in Figure S7. In the EMA method, optical transmittances of the psWGPs are calculated by approximating each structure to an anisotropic uniaxial thin film according to the air gap of the Al structure. FDTD results are used as a reference. A negligible discrepancy between the simulation results attained by using FDTD and EMA is observed, which are in good agreement with the measured results.

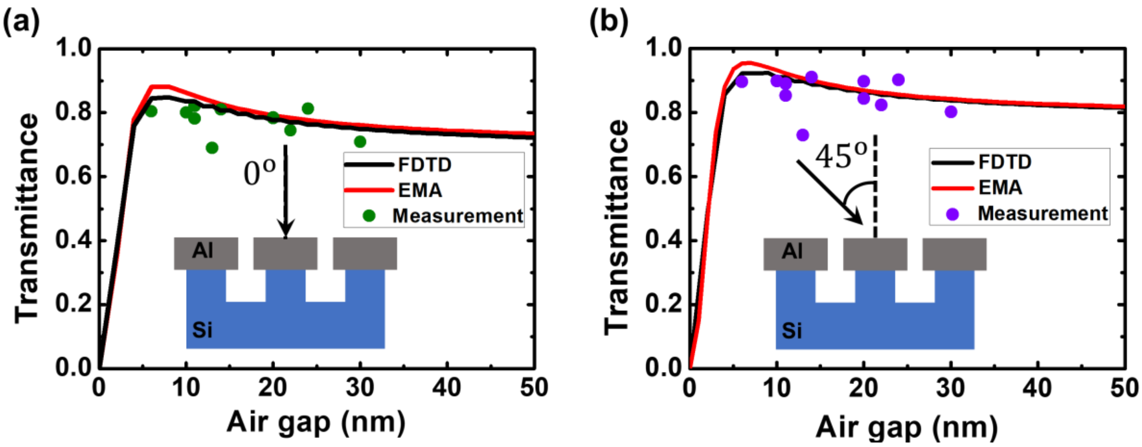

Figure S8. TM transmittances of the psWGP structure, calculated by utilizing FDTD (black) and EMA (red) methods, as a function of the air gap at (a) 0˚ and (b) 45˚. Measured data are also included.

S6. Detailed calculation of admittance trajectory example.

Complex admittance Y of the [air|Al nanowire (EMA Al)|Si nano-pattern (EMA Si)|Si] structure at normal incidence for TM wave of 4000 nm wavelength in Fig. 4(c) in the main text is calculated as the thickness increases and the trajectory of Y in the complex admittance diagram is presented explicitly in Figures S9 (a) and (b). Complex refractive index of EMA Al layer at 6-nm air gap at 4000 nm wavelength can be obtained as $N_{x,\,EMA\,Al} = 4.0890 - i0.0016$ from either Figure 4(b) in the main text or Figure S7 (a) in SI, and the effective index of EMA Si layer at 50-nm air gap is $N_{x,\,EMA\,Si} = 1.3574$ from Figure S7 (b) in SI. The optical phase thickness in Eq (3) of the main text can be written as $\delta = \frac{2\pi}{\lambda} N_x h$ at normal incidence, and the physical thicknesses of EMA Al-nanowire layer and EMA Si-pattern layer are $h_{EMA\,Al} = 44\text{ nm}$ and $h_{EMA\,Si} = 140\text{ nm}$, respectively.

Admittance Y of an EMA Si-pattern layer starts from that of Si substrate ($Y_{sub} = 3.42$, point 'a') as shown in Figure S9 (a). As the optical thickness increases, the trajectory of Y can be calculated by two equations of $\begin{bmatrix} B \\ C \end{bmatrix} = [M_{EMA\,Si}] \begin{bmatrix} 1 \\ Y_{sub} \end{bmatrix}$ and $Y = \frac{C}{B}$. At the thickness of $h_{EMA\,Si} = 140$ nm, it arrives at point 'b' of $Y_b = \left(\frac{C}{B}\right)_b = 2.34 - i1.40$.

Similarly, admittance Y of an EMA Al nanowire layer starts from Y_b as shown in Figure S9 (b). As the optical thickness increases, the trajectory of Y is calculated by using $\begin{bmatrix} B \\ C \end{bmatrix} = [M_{EMA\,Al}] \begin{bmatrix} 1 \\ Y_b \end{bmatrix}$ with $Y = \frac{C}{B}$ and arrives at point 'c' of $Y_c = \left(\frac{C}{B}\right)_c = 2.06 - i0.49$ at $h_{EMA\,Al} = 140$ nm.

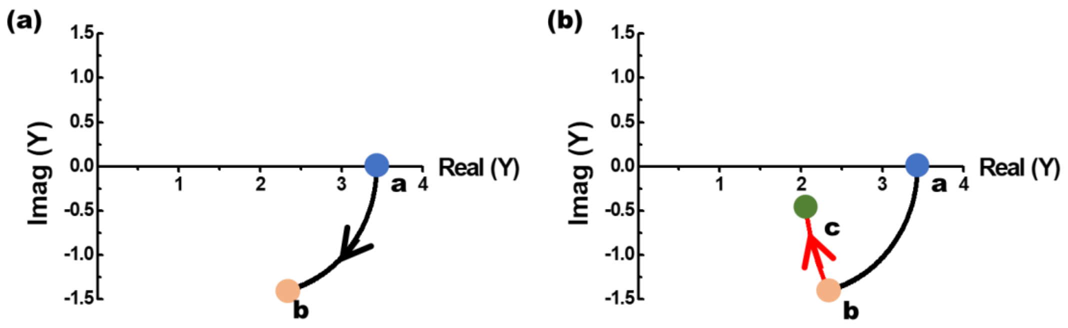

Figure S9. (a) Admittance diagram of EMA Si-pattern layer of 140-nm thickness at 4000 nm wavelength. The starting point is 'a' (3.42, 0) of Si substrate admittance, the black solid line is an admittance trajectory of EMA Si-pattern layer calculated by using Eqs. (3)~(5) in the main text, and the admittance arrives at 'b' of Y_b =(2.34, -1.40) at $h_{EMA\,Si}$ = 140 nm, and (b) admittance diagram of an EMA Al-nanowire layer starts from 'b' (2.34, -1.40), the red solid line is an admittance trajectory of the 44-nm-thick EMA Al-nanowire, and the final admittance of [air|Al-nanowires|Si-nanopatterns|Si substrate] arrives at 'c' of Y_c =(2.04, -0.49) at $h_{EMA\,Al}$ = 140 nm.

S7. Dispersion relation of the wire grid polarizer

From Equations (3) - (6) in the main text, the amplitude transmission coefficient of [air|EMA Al-nanowires|substrate] structure can be derived as

$$t = \frac{2Y_0}{Y_0 B + C} = \frac{2Y_0}{(Y_0 + Y_S)\cos\delta + i\,(\eta + \frac{Y_0 Y_S}{\eta})\sin\delta}. \tag{S1}$$

In case of the symmetric structure of $Y_0 = Y_s$ in [air|EMA Al-nanowires|air], the dispersion

relation can be obtained when t diverges, i.e.,

$$\left(1-\tan^2\frac{\delta}{2}\right)+i\frac{\eta^2+Y_0^2}{\eta Y_0}\tan\frac{\delta}{2}=0. \quad \text{(S2)}$$

Since two solutions can be found in Eq. (S2), the dispersion can be expressed as

$$\begin{cases} i\tan\frac{k_z h}{2}=-\frac{\varepsilon_x k_{iz}}{k_z} & (even\ solution) \\ i\cot\frac{k_z h}{2}=\frac{k_z}{\varepsilon_x k_{iz}} & (odd\ solution) \end{cases} \quad \text{(S3)}$$

where $\delta=k_z h$, $\eta=\varepsilon_x\frac{k_0}{k_z}$, $Y_0=\frac{k_0}{k_{iz}}$, and $k_0=\frac{\omega}{c}$. From the phase matching condition of wavevectors in the x-direction, the diffraction equation can be written as $k_{dx}=k_{ix}=k_0 n_i\sin\theta_i+n\frac{2\pi}{d}$, where k_{dx} and k_{ix} are the diffracted and incident wavevector in x-direction, respectively, n is the order of diffraction, and d is the period. In the long wavelength limit of $d\ll\lambda$, k_{ix} can become the pure imaginary, $k_{iz}=-i\sqrt{k_{ix}^2-k_0^2}$, if k_{ix} is larger than k_0 at the order of n. The optical phase thickness can be approximated as $\delta=k_z h=n_{eff}k_0 h\approx\frac{\omega}{c}h$, if the effective index (n_{eff}) of the fundamental waveguide mode in the narrow air-gap is assumed as 1. Then, the dispersion of WGP in air for even mode can be expressed as [RS4, RS5]

$$\tan\left(\frac{\omega}{c}\frac{h}{2}\right)=\varepsilon_x\frac{\sqrt{k_{ix}^2-\left(\frac{\omega}{c}\right)^2}}{\frac{\omega}{c}}, \quad \text{(S4)}$$

which is the same as Eq. (7) in the main text.

References

RS1. Yamada, I. *et al.* Transmittance enhancement of a wire-grid polarizer by antireflection coating. *Appl. Opt.* **48**, 316-320 (2009)

RS2. Kim, W. et al. Facile fabrication method for broadband Al wire grid polarizers using nanoimprint lithography and oblique angle deposition. *Sci. Adv. Mater.* **10**, 660-664 (2018).

RS3. Yeon, J. et al. High throughput ultralong (20 cm) nanowire fabrication using a wafer-scale nanograting template. *Nano Let.* **13**, 3978-3984 (2013)

RS4. Shen, J. T., Catrysse, P. B. & Fan, S. Mechanism for designing metallic metamaterials with a high index of refraction. *Phy. Rev. Lett.* **94**, 197401 (2005).

RS5. Li, Z. et al. High-Contrast Gratings based Spoof Surface Plasmons. *Sci. Rep.* **6**, 21199 (2016).
